# An interim report on the investigator-initiated phase 2 study of pembrolizumab immunological response evaluation (INSPIRE)

**DOI:** 10.1186/s40425-019-0541-0

**Published:** 2019-03-13

**Authors:** Derek L. Clouthier, Scott C. Lien, S. Y. Cindy Yang, Linh T. Nguyen, Venkata S. K. Manem, Diana Gray, Michael Ryczko, Albiruni R. A. Razak, Jeremy Lewin, Stephanie Lheureux, Ilaria Colombo, Philippe L. Bedard, David Cescon, Anna Spreafico, Marcus O. Butler, Aaron R. Hansen, Raymond W. Jang, Sangeet Ghai, Ilan Weinreb, Valentin Sotov, Ramy Gadalla, Babak Noamani, Mengdi Guo, Sawako Elston, Amanda Giesler, Sevan Hakgor, Haiyan Jiang, Tracy McGaha, David G. Brooks, Benjamin Haibe-Kains, Trevor J. Pugh, Pamela S. Ohashi, Lillian L. Siu

**Affiliations:** 1 Princess Margaret Cancer Centre, University Health Network, Toronto, Canada; 2 Department of Immunology, University of Toronto, Toronto, Canada; 3 Department of Medical Biophysics, University of Toronto, Toronto, Canada; 4 Division of Medical Oncology and Hematology, Department of Medicine, University of Toronto, Toronto, Canada; 5 Joint Department of Medical Imaging, University Health Network, Toronto, Canada; 6 Department of Biostatistics, Princess Margaret Cancer Centre, Toronto, Canada; 7 Department of Computer Science, University of Toronto, Toronto, Canada; 8 Ontario Institute of Cancer Research, Toronto, Canada; 9 Vector Institute, Toronto, ON Canada; 10 Division of Medical Oncology and Hematology, Princess Margaret Cancer Centre, 700 University Ave, Toronto, ON M5G 1Z5 Canada

**Keywords:** Biomarkers, Mechanisms of sensitivity, Mechanisms of resistance, Immunotherapy, Immunology, Drug mechanisms

## Abstract

**Background:**

Immune checkpoint inhibitors (ICIs) demonstrate unprecedented efficacy in multiple malignancies; however, the mechanisms of sensitivity and resistance are poorly understood and predictive biomarkers are scarce. INSPIRE is a phase 2 basket study to evaluate the genomic and immune landscapes of peripheral blood and tumors following pembrolizumab treatment.

**Methods:**

Patients with incurable, locally advanced or metastatic solid tumors that have progressed on standard therapy, or for whom no standard therapy exists or standard therapy was not deemed appropriate, received 200 mg pembrolizumab intravenously every three weeks. Blood and tissue samples were collected at baseline, during treatment, and at progression. One core biopsy was used for immunohistochemistry and the remaining cores were pooled and divided for genomic and immune analyses. Univariable analysis of clinical, genomic, and immunophenotyping parameters was conducted to evaluate associations with treatment response in this exploratory analysis.

**Results:**

Eighty patients were enrolled from March 21, 2016 to June 1, 2017, and 129 tumor and 382 blood samples were collected. Immune biomarkers were significantly different between the blood and tissue. T cell PD-1 was blocked (≥98%) in the blood of all patients by the third week of treatment. In the tumor, 5/11 (45%) and 11/14 (79%) patients had T cell surface PD-1 occupance at weeks six and nine, respectively. The proportion of genome copy number alterations and abundance of intratumoral 4-1BB+ PD-1+ CD8 T cells at baseline (*P* < 0.05), and fold-expansion of intratumoral CD8 T cells from baseline to cycle 2–3 (*P* < 0.05) were associated with treatment response.

**Conclusion:**

This study provides technical feasibility data for correlative studies. Tissue biopsies provide distinct data from the blood and may predict response to pembrolizumab.

**Electronic supplementary material:**

The online version of this article (10.1186/s40425-019-0541-0) contains supplementary material, which is available to authorized users.

## Background

Immune checkpoint blockade has shown unprecedented success in treating a variety of cancers [1]. Despite extensive efforts in translational research and clinical trials involving ICIs, there remain gaps in elucidating the mechanisms of response or resistance with these agents.

The objective response rates to anti-programmed death protein 1/ligand 1 (anti-PD-1/−L1) agents vary widely by tumor type, but overall average approximately 20% among tumor types with demonstrated efficacy [2]. The challenge is to identify the approximately one in five patients who are most likely to respond to single-agent ICIs or select those who may require more aggressive combination therapies. Carefully designed biomarker studies may generate clinically meaningful tests to select patients and potentially avoid unnecessary toxicities and reduce cost per life-year saved with ICIs. Predictive and mechanistic biomarker studies may also inform how to rationally combine ICIs with other modalities or agents and when to prioritize ICI use in the treatment sequence.

There are still very few validated predictive biomarkers (reviewed in [3]) and the mechanisms of sensitivity and resistance to ICIs are not completely understood. To date, the greatest focus has been on the degree of expression of PD-L1 on tumor and infiltrating immune cells, tumor infiltrating lymphocytes (TILs), tumor mutational burden (TMB), neoantigen load [4, 5], and T cell receptor (TCR) clonality (reviewed in [1, 3, 6]). While the most promising biomarker studies to date come from randomized, prospective trials, these studies are often limited in the scope of genomic and immune correlatives because they use archival specimens, and do not include on-treatment biopsies to obtain a mechanistic understanding of the dynamic antitumor immune response. The INSPIRE trial (NCT02644369) was carefully designed to leverage integrated genomic and immune parameters from freshly processed tissue biopsies and peripheral blood before, during, and after treatment with the anti-PD-1 monoclonal antibody, pembrolizumab. The primary objective of INSPIRE was to determine whether genomic and/or immune biomarkers were associated with response to pembrolizumab. In addition to informing the development of future biomarker studies and improving our mechanistic understanding of ICIs, this study provides a prioritized and optimized tissue and blood processing workflow and identifies practical issues to consider when designing in-depth correlative studies across different histologies.

## Materials and methods

For additional details related to study methods, please see Additional file 1: Supplementary methods.

### Study design

The INvestigator-initiated Phase 2 Study of Pembrolizumab Immunological Response Evaluation (INSPIRE) is a single-centre study approved by the Research Ethics Board at the Princess Margaret Cancer Centre and registered at https://clinicaltrials.gov/ct2/show/NCT02644369 and is being conducted in accordance with the principles of Good Clinical Practice, the provisions of the Declaration of Helsinki, and other applicable local regulations.

### Patient selection and drug administration

INSPIRE is a basket study with five cohorts: A, squamous cell carcinoma of the head and neck (SCCHN); B, triple negative breast cancer (TNBC); C, high-grade serous ovarian cancer (HGSC); D, metastatic melanoma (MM); E, mixed advanced solid tumors (MST) (Additional file 2: Table S1). The first 80 patients enrolled are described in the current report. Male and female subjects who were 18 years or older, had a histological or cytological diagnosis of protocol specified solid malignancies, and were refractory to or lacked appropriate standard therapies, were eligible. Pembrolizumab was administered intravenously at a fixed dose of 200 mg over 30 min once every three weeks.

### Tumor biopsies and blood collection

Mandatory tumor biopsies were collected at baseline (within 28 days of study treatment), on-treatment during the final week of the second or third cycle of pembrolizumab (week six or week nine, respectively; the time of the on-treatment biopsy was changed from week nine to week six to increase the proportion of patients who remained on study for the on-treatment biopsy). For patients with a confirmed partial or complete response (PR, CR) or prolonged stable disease (> 4 months), a third optional biopsy was taken at progression (Additional file 3: Figure S1). In 6/80 (7%) cases where the pre-treatment formalin-fixed paraffin-embedded (FFPE) tumor tissue core processed from fresh biopsy contains no tumor cells (by pathology assessment), archival specimens from standard of care procedures performed before the start of INSPIRE were used for immunohistochemistry (IHC). Biopsies were either excisional or image-guided needle biopsies. Every effort was made to ensure that the on-treatment biopsies were taken from the same site as the baseline biopsy.

### IHC

For screening biopsies only, the FFPE blocks were used for PD-L1 IHC (clone 22C3) on 4–5 μm sections mounted on positively charged ProbeOn slides (QualTek, Goleta, CA). QualTek provided a modified proportion score (MPS) indicating the proportion of PD-L1-expressing tumor cells and mononuclear inflammatory cells within tumor nests. For detailed information on IHC methods or MPS calculation, see Additional file 1: Supplementary methods.

### Tissue processing

Pooled core biopsies or tissue samples were minced into 2-4 mm^3^ fragments and digested with the gentle MACS dissociator (Miltenyi, Catalog #130–093-235) and the human tumor dissociation kit (Miltenyi, Catalog #130–095-929).

### Whole exome sequencing (WES)

DNA extracted from digested tumor biopsies were sequenced with Illumina sequencing at the Princess Margaret Genomic Centre and the Princess Margaret - Ontario Institute of Cancer Research Translational Genomics Laboratory (PM-OICR TGL) in Toronto, Canada. Exonic regions were enriched using Agilent SureSelectXT (Santa Clara, CA) target enrichment and hybridization to Agilent SureSelect Human All Exon V5 + UTRs baits. Pooled libraries were normalized to 10 nM and sequenced using either HiSeq2000 or HiSeq2500 following manufacturer’s protocols. Paired-end 125 bp reads were generated to target median coverage of 250X for tumor samples and 50X for control blood DNA. De-multiplexed WES reads were aligned to the human genome reference GRCh38 using the Burrows-Wheeler Alignment tool v0.7.12 followed by PCR duplicate reads removal and Indel re-alignment as described by the Genome Analysis ToolKit Best Practices for Somatic SNV Discovery in Whole Genome and Exome Sequencing [7]. Somatic mutations were identified using MuTect2 [8] with default settings. Somatic genome copy number alterations were detected using Sequenza [9]. For detailed information on calculations of tumor mutation burden and percent genome alteration, see Additional file 1: Supplementary methods.

### Flow cytometry

PBMCs or tumor single cell suspensions were stained for immune markers of interest. Data were acquired using a 5-laser LSR Fortessa X-20 (BD, Mississauga, Ontario, Canada). Immunophenotyping data were analyzed using FlowJo v10 (Treestar, Ashland, Oregon, USA). For detailed information on the optimized flow cytometry panels, please refer to Additional file 2: Table S2.

### Data analysis and statistics

Group differences were examined using Student’s t test or one-way ANOVA for continuous measures. Time to progression was calculated from the start date of treatment to the date of progression; patients alive without progression were censored on the date of last follow-up. Progression rate was calculated using the cumulative incidence function method with death treated as a competing risk [10]. All tests were two-sided with *P* < 0.05 considered to be statistically significant. All analyses were performed using the SAS software 9.3 or GraphPad Prism 7 (San Diego, California, USA). Concordance index (c index) was used to compute the association of each variable with the clinical response to pembrolizumab [11]. Analysis was limited to the variables with measurements for at least five patients per RECISTv1.1 response category. Nominal *p*-values were calculated using the Noether approach [12], and further corrected for multiple testing using the false discovery approach [13]. The cut-off value for significance was a false discovery rate (FDR) of 5%, and a Kruskal Wallis test [14] was used to compare multiple groups.

## Results

From March 21, 2016 to June 1, 2017, 80 patients were accrued: eight SCCHN, 13 TNBC, 21 HGSC, 10 MM, and 28 MST patients. All patients were ECOG 0 or 1, and 79% of patients were treated with at least one prior systemic therapy. A range (0–100%) of baseline PD-L1 IHC MPS were observed, including 51% of patients with baseline tumor PD-L1 MPS of 0 (Additional file 2: Table S1).

The median (range) follow-up time was 4.1 (0–13.8) months, and the median number of cycles of pembrolizumab delivered in the SCCHN, TNBC, HGSC, MM and MST cohorts were: 4, 3, 3, 10 and 5, respectively. The objective response rates in these five cohorts were: 2/8 (25%), 1/13 (8%), 0/21 (0%), 6/10 (60%) and 4/28 (15%) with no CRs (Additional file 2: Table S3), and the median time-to-progression was: 3.4, 2.0, 2.8, not reached and 3.5 months, respectively (Additional file 2: Table S3, Additional file 4: Figure S2). Overall, pembrolizumab was well tolerated with the most common grade 1–2 adverse events (AEs) being fatigue (38%), diarrhea (21%) and nausea (16%) (Additional file 2: Table S4). Grade 3–4 adverse events were uncommon with the most frequent being fatigue (3%) and pneumonitis (3%) (Additional file 2: Table S4). Fifty of 57 (88%) patients who discontinued treatment did so due to disease progression. Four (21%) HGSC patients discontinued due to AEs, side effects, or complications (Additional file 2: Table S3).

### Peripheral blood and biopsy collection

**Fig. 1:**
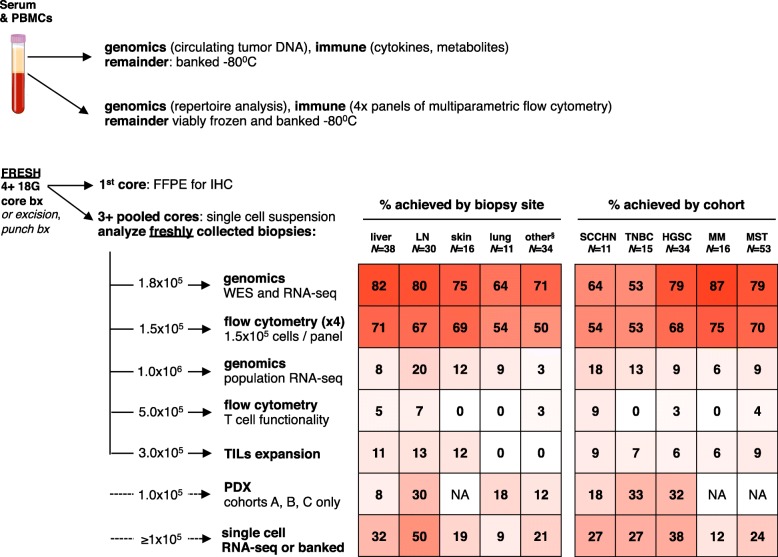
Prioritization of correlative samples and feasibility of assays by histology and biopsy site. Note: six samples excluded due to technical errors between May 1, 2017 and June 1, 2017. ^§^most common sites included peritoneal mass (12), abdominal wall (5), chest wall (3), omentum (3), other (11). FFPE, formalin-fixed paraffin embedded; HGSC, high grade serous ovarian cancer; IHC, immunohistochemistry; LN, lymph node; PDX, patient derived xenograft; MM, metastatic melanoma; MST, mixed advanced solid tumors; SCCHN, squamous cell carcinoma of the head and neck; TIL, tumor infiltrating lymphocyte; TNBC, triple negative breast cancer; WES, whole exome sequencing

Ambitious goals were set to maximize the number of assays to allow in-depth characterization of each biopsy tissue collected (Fig. 1). However, in many instances, there were not enough cells from fresh tissue biopsies to achieve all of the exploratory research objectives. From the first 80 patients enrolled, 52/80 (65%) had paired biopsies. 7/80 (9%) were not yet collected, and 21/80 (26%) did not have on-treatment biopsies collected because they discontinued due to disease progression or toxicity before collection was possible. Overall, 33/80 (41%) of patients had evaluable baseline and 17/52 (33%) had evaluable paired baseline and on-treatment biopsies for genomic and at least one flow cytometry panel for basic immunophenotyping.

For sequential biopsies in INSPIRE, every effort was made to biopsy the same lesion within each case; 43/52 (83%) of the patients had the same lesions biopsied at baseline and on-treatment. To reduce the likelihood of capturing spatial bias from a single biopsy that was not representative of the entire tumor mass [15], independent needle, core, or excisional biopsies were pooled prior to tissue digestion to obtain an average sampling of the heterogeneity of cells within the tumor. To inform future studies, biopsy quality was evaluated by histological group and biopsy site. Liver, lymph node, cutaneous, and lung biopsies provided 1.94, 2.93, 3.49, and 2.76 × 10^5^ cells/core, respectively (Table 1). Similar numbers of cores were collected from the different biopsy sites (Table 1). Although some tumor types provided more cells per core (Table 1), this did not always result in better technical outcomes (Fig. 1).

**Table 1:** Technical feasibility and efficiency of correlative sample analysis efficiency by histology and biopsy site^a,b^

TOTAL *N* = 129			total # of cell recovered, mean (range) × 10^6^	# of cores, mean (range)	# of cells per core, mean (range) × 10^6^
DATA BY BIOPSY SITE					
liver	Core	*N* = 36	0.876 (0.057–8.78)	4.42 (2–8)	0.194 (0.016–1.46)
	Exn	*N* = 2	1.76 (1.28–2.25)		
LN	Core	*N* = 29	1.00 (0.049–4.07)	3.66 (1–6)	0.293 (0.024–1.36)
	Exn	*N* = 1	10.8		
skin	Core	*N* = 9	1.19 (0.011–6.70)	4.78 (3–11)	0.349 (0.004–2.23)
	Exn	*N* = 7	1.46 (0.021–3.35)		
lung	Core	*N* = 10	1.22 (0.001–6.50)	5.00 (3–8)	0.276 (0.002–1.63)
	Exn	*N* = 1	0.073		
other^b^	Core	*N* = 33	0.49 (0.012–1.65)	4.21 (1–8)	0.121 (0.002–0.40)
	Exn	*N* = 1	14.4		
DATA BY HISTOLOGY					
SCCHN	Core	*N* = 10	1.92 (0.009–8.78)	3.90 (1–6)	0.42 (0.002–1.63)
	FNA	*N* = 1	0.07		
TNBC	Core	*N* = 13	0.57 (0.027–1.87)	4.54 (2–8)	0.151 (0.007–0.62)
	Exn	*N* = 2	0.99 (0.035–1.95)		
HGSC	Core	*N* = 33	0.72 (0.013–4.07)	3.94 (1–8)	0.211 (0.003–1.36)
	Exn	*N* = 1	14.4		
MM	Core	*N* = 12	0.54 (0.012–1.13)	4.42 (1–8)	0.120 (0.002–0.28)
	Exn	*N* = 4	1.58 (0.021–3.35)		
MST	Core	*N* = 49	0.87 (0.011–6.70)	4.41 (2–11)	0.220 (0.004–2.23)
	Exn	*N* = 4	4.05 (1.28–10.8)		

^a^six samples excluded due to technical errors between May 1, 2017 and June 1, 2017

^b^most common sites included peritoneal mass (12), abdominal wall (5), chest wall (3), omentum (3), others (11)

*Exn* Excision, *FNA* fine needle aspirate, *LN* lymph node

By biopsy site, liver, lymph node and cutaneous biopsies provided 82, 80, and 75% of samples for DNA and RNA sequencing and 71, 67, and 69%, respectively of samples for at least one panel of multiparameter flow cytometry (Fig. 1). In contrast, lung, chest and abdominal wall biopsies yielded less evaluable tissue with each pass and these biopsies had lower cellularity and yielded less DNA and RNA and high-quality cell suspensions that were suitable for flow cytometric evaluation. By histology, MM, HGSC, MST cohorts yielded the most samples for sequencing and flow cytometry (Fig. 1). SCCHN and TNBC cohorts did not provide as many high quality samples. 8/11 (73%) of SCCHN biopsies were from the lung, which generally did not yield high quality tissue for correlates. The TNBC cohort was also challenging from a technical perspective regardless of biopsy site: 6/15 (40%) LN, 4/15 (27%) liver, 2/15 (13%) lung, 2/15 (13%) cutaneous, and 1/15 (7%) chest wall; this may reflect features that are intrinsic to TNBC.

### Activation-induced T cell co-signaling molecule expression is significantly higher in the tumor than in the blood

**Fig. 2:**
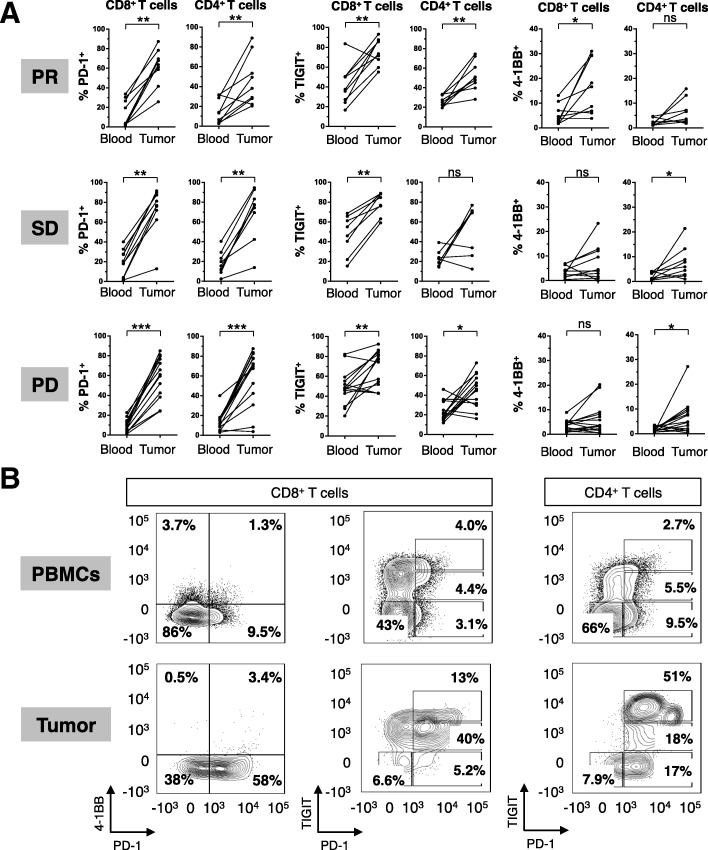
Discordant T cell phenotypes in the tumor compared to circulating lymphocytes. (**a**) Summary of paired tumor and PBMC CD4 and CD8 T cell phenotyping for 4-1BB, TIGIT, and PD-1 at baseline. (**b**) Representative FACS of paired peripheral blood mononuclear cells and tumor biopsies. *P < 0.05, ** *P* < 0.01, *** *P* < 0.001

The profile of T cell co-signaling molecule expression was distinct between T cells from the peripheral blood compared to those from the tumor at baseline. Intratumoral T cells had significantly higher expression of the T cell activation markers PD-1, TIGIT, 4-1BB (Fig. 2a), with representative staining shown (Fig. 2b). This trend was observed in patients across all histological cohorts and RECIST response categories. Similar trends were observed in the level of expression, as assessed by median fluorescence intensity (MFI) of PD-1, 4-1BB, and TIGIT on both CD8 and CD4 T cells (Additional file 5: Figure S3).

### Pembrolizumab occupies T cell PD-1 in the blood more rapidly than in the tumor

**Fig. 3:**
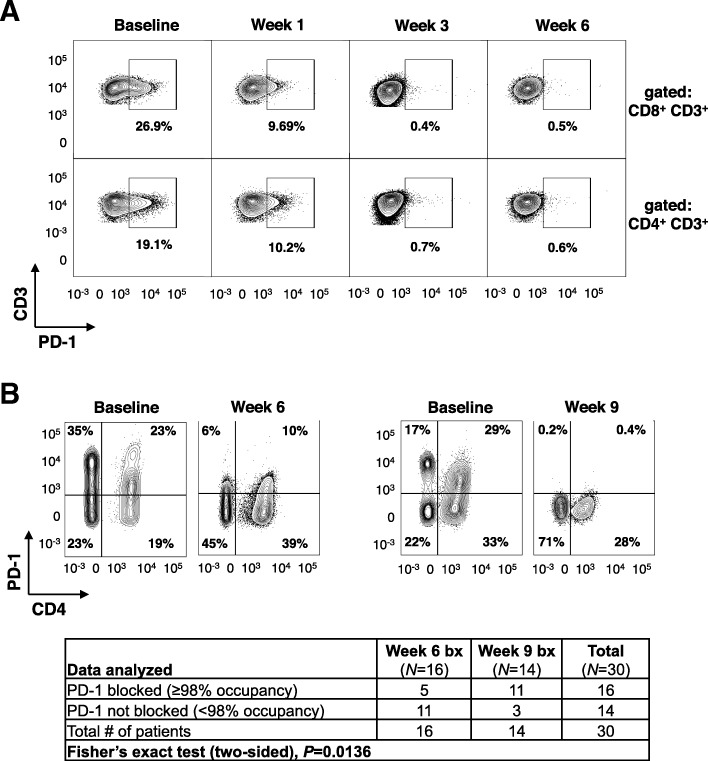
PD-1 is not detectable on peripheral blood T cells or tumor-infiltrating T cells after six and nine weeks of pembrolizumab, respectively. (**a**) Representative PD-1 staining of CD4 and CD8 T cells from peripheral blood at baseline and the first six weeks of pembrolizumab. (**b**) Representative FACS from patients with PD-1 blocked by week six and week nine of treatment. Gated on total CD3+ lymphocytes in tumor biopsies. Clone of anti-PD-1 used for flow cytometry was EH12.2H7

To understand the kinetics of pembrolizumab binding its target in vivo, PD-1, in the peripheral blood and the tumor, PD-1 expression was detected by flow cytometry at discrete time points after treatment with pembrolizumab using clone EH12.2H7, which is blocked by pembrolizumab. PD-1 expression was detectable at baseline in both PBMC and tumor samples. However, PD-1 was blocked by at least 98% of peripheral blood CD4 and CD8 T cells in 75/80 (94%) of patients after three weeks of treatment with pembrolizumab (Fig. 3a and Additional file 6: Figure S4A). In contrast, in the 16 patents with evaluable biopsies after six weeks of pembrolizumab treatment, 11/16 (69%) patients had detectable PD-1, and 3/14 (21%) patients had detectable PD-1 after nine weeks of pembrolizumab treatment (*P* = 0.0136; Fig. 3b). Note that the week six and nine biopsies evaluated PD-1 expression in different patients. *PDCD1* mRNA levels were similar at baseline and on-treatment tumor biopsies, ruling out the possibility of reduced PD-1 expression due to *PDCD1* transcript downregulation (Additional file 6: Figure S4B). Taken together, these data suggest that PD-1 blockade occurs more rapidly in the peripheral blood and takes approximately six to nine weeks to mask PD-1 on T cells in the tumor.

### Tumor and blood genomic and immune parameters correlate with clinical response to pembrolizumab

**Fig. 4:**
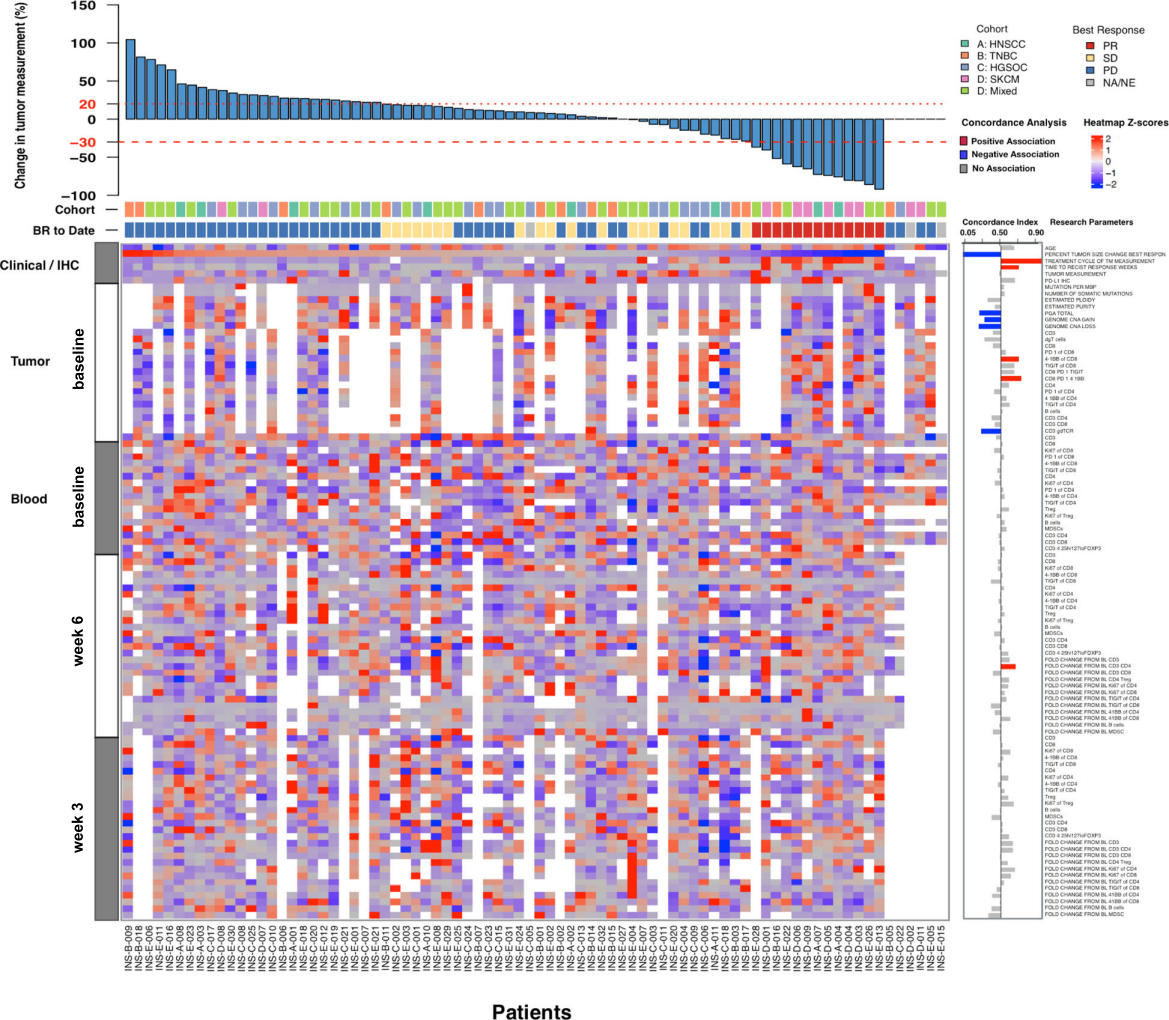
Clinical, genomic and immune correlates in INSPIRE. A composite display of 107 clinical, genomic, and immune correlates for each patient (per column) at baseline and on-treatment tumor and peripheral blood samples, sorted in order of decreasing percentage change in tumor measurement from baseline. Cohort and best RECIST 1.1 response for each patient is shown in the color track below the bar plot. Data for each row is z-score normalized. C index for variable is shown (right), with statistically significant associations highlighted in blue or red (respectively, negative and positive associations with favorable clinical response). PGA, percent genome altered

A major collaborative effort was made to collect, integrate, and select 104 clinical, genomic, and immune variables for the first 80 patients enrolled in INSPIRE (Fig. 4). Using a FDR < 5%, seven variables significantly associated with clinical response (Fig. 4; two factors were controls: percent change of tumor measurements from baseline and time to response). Univariable association between the collected variables and clinical response categories was calculated using the c index for 74 patients. Very few combinatorial predictive biomarkers have been identified, but there are several individual factors that are positively or negatively associated with clinical response to pembrolizumab.

PD-L1 IHC as a continuous variable at baseline did not meet the threshold for a positive correlation with clinical response; however, it trended toward a positive correlation with outcome across all tumor cohorts (Fig. 4). It was recently reported that PD-L1 expression measured from a single biopsy lacked sensitivity to reflect the PD-L1 expression of the whole tumour tissue, suggesting that our observation may be affected by intratumor heterogeneity [16]. Neither non-synonymous mutation burden nor total number of non-synonymous somatic mutations correlated with clinical response to pembrolizumab (Fig. 4).

**Fig. 5:**
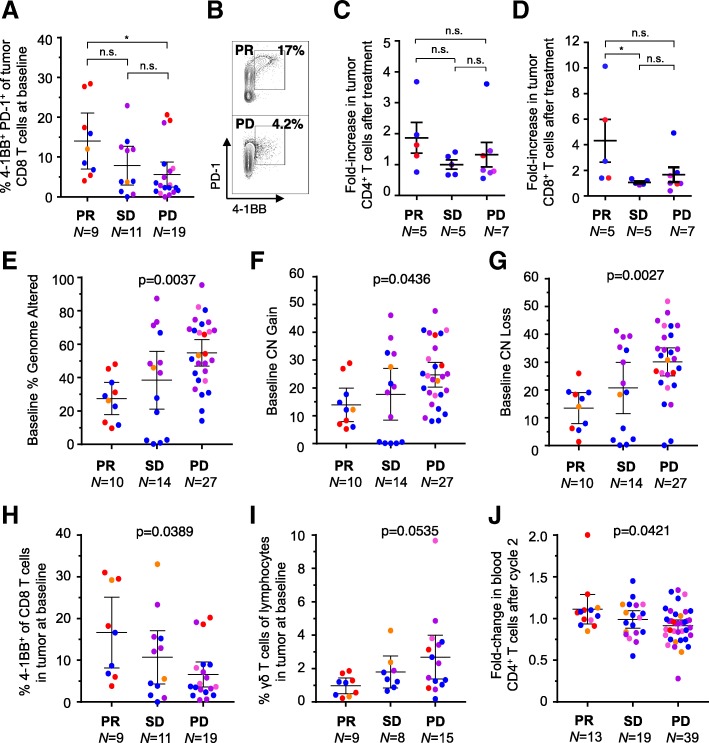
Genomic and immune correlates in INSPIRE. (**a**) The frequency of tumor-infiltrating 4-1BB+ PD-1+ CD8 T cells at baseline from fresh tumor biopsies, with representative median flow cytometry staining (**b**) from patients who achieved a PR or PD as their best response. (**c,d**) Flow cytometry-based assessment of fold-change in CD4 and CD8 T cells between fresh baseline and on-treatment biopsies. (**e**-**j**) Distribution of the measurements with respect to response categories for each factor identified as significantly predictive. *P* values were determined using the Kruskal-Wallis test. Orange, SCCHN; Pink, TNBC; Purple, HGSC; Red, MM; Blue, MST

Factors that associated with clinical response were then interrogated by RECIST1.1 best responses (Fig. 5). From the 39 patients with baseline tumor samples that were evaluable for at least one flow cytometry immunophenotyping panel, the frequency of tumor infiltrating 4-1BB + PD-1+ CD8 T cells at baseline was positively associated with clinical response. Patients with a confirmed PR had approximately 2-fold more 4-1BB+ PD-1+ CD8 T cells at baseline than patients with a best response of PD (*P* < 0.05) or SD (n.s.) (Fig. 5a-b).

Fold-changes in immune parameters from the tissue were assessed in 17 patients with paired biopsies and sufficient material for immune correlates. The fold-increase in CD8, but not CD4 T cells from before to after therapy was significantly positively associated with response to pembrolizumab in patients with a best response of SD (SD, *p* < 0.05; PD, n.s.; Fig. 5c-d). Other factors that significantly associated with clinical response to pembrolizumab treatment include percent of genome altered (PGA), gene copy number (CN) gains and losses, percent 4-1BB^+^ of CD8^+^ T cells, percent of infiltrating γδ T cells, and fold-change from baseline to on-treatment in peripheral blood CD4^+^ T cells (Fig. 5e-j). Correlative data were also stratified by tumor type and the trends were maintained for all of the seven parameters that significantly associated with clinical response except for fold-change in CD4 T cells in the blood from baseline to cycle three (Additional file 2: Table S5).

Some biomarkers were correlated with each other, such as baseline tumor PD-1+ 4-1BB+ CD8 T cells and TMB (Additional file 7: Figure S5), especially in the MM cohort. There was a not a significant correlation between PD-L1 and TMB, similar to previous reports [17], suggesting that these biomarkers may independently predict response to pembrolizumab.

## Discussion

This study provides an interim report from a phase 2 trial of pembrolizumab in a variety of solid tumors and preliminary translational data. PD-L1 status and TMB appear to enrich for response to ICIs, but application of these biomarker results and comparison against other studies in the literature are complicated by the use of different antibody clones, cell populations included or excluded during scoring, inter-observer variability, and thresholds for positivity. INSPIRE was not powered to discover and validate specific predictive biomarkers of ICIs, but rather, it was a hypothesis-generating study to comprehensively evaluate the baseline and dynamic changes in the genomic and immune landscape of several solid tumor histologies following treatment with pembrolizumab.

Some of the results generated from this study are largely technical in nature, and are provided to inform investigators who intend to perform similar translational analyses (Fig. 1). Previous studies have shown that peripheral blood and tumor-infiltrating T cells express differing levels of PD-1 [18], and here we show that this is also true for other T cell co-signaling molecules including 4-1BB and TIGIT (Fig. 2). Taken together, these studies demonstrate that fresh tumor biopsies are informative and feasible in many cases and peripheral blood immune biomarkers may not be an appropriate surrogate for ongoing immune responses in the tumor. This is an important point for future investigators to consider when designing correlative studies.

A handful of studies have identified possible biomarkers of response to ICIs in peripheral blood such as high lymphocyte or eosinophil counts, low baseline neutrophil, regulatory T cell or myeloid-derived suppressor cells (MDSC) counts, TCR gene richness and evenness at baseline [3, 19], and a high baseline frequency of classical monocytes [20]. In our study, we did not find any trends that are predictive of response to pembrolizumab based on peripheral blood samples taken at baseline; however, fold-expansion of CD4 T cells from baseline to week three was higher among responders (Fig. 5j). New promising indicators of response to ICIs, such as the post-pembrolizumab fold-change of Ki67+ PD-1+ CD8 T cells relative to tumor burden [21], CD28 expression on CD8 T cells [22] will be considered in future analyses.

TMB did not correlate with response in this study, but this was not unexpected given the heterogeneity of tumor types tested. PD-L1 MPS values by IHC and T cell PD-1 expression by flow cytometry trended toward enriching for response to pembrolizumab, but were overlapping between responders and non-responders. The addition of 4-1BB to the biomarker panel further separated responders from non-responders, better than T cell PD-1 as a single variable. 4-1BB is a co-stimulatory member of the tumor necrosis factor receptor superfamily and is upregulated on antigen-activated T cells [23]. The frequency of 4-1BB+ PD-1+ CD8 T cells in the tumor at baseline demonstrated a statistically significant separation of responders from non-responders (Fig. 5a-b), and validation of this finding using multicolor IHC is planned. Given that 4-1BB is induced by TCR signalling, it is interesting to speculate that the presence of 4-1BB+ PD1+ CD8 T cells may be indicative of an ongoing tumor-specific T cell response, which could be augmented by pembrolizumab. It has been previously shown in CD8 T cells freshly isolated from excised melanoma lesions that PD-1 expression identified tumor-reactive CD8 TILs [24]. While 4-1BB could also be used to enrich for tumor-reactive CD8 TILs, PD-1 expression more comprehensively captured the clonally expanded repertoire of tumor-reactive CD8 T cells. However, it has also been shown in ovarian cancer and melanoma that 4-1BB expression better identifies tumor-reactive CD8 TILs; 4-1BB+ CD8 TILs responded to peptide stimulation of a HLA-matched cancer cell line, while 4-1BB- CD8 T cells did not [25]. It is known that PD-1 is more broadly expressed on tumor and tumor-unrelated virus specific CD8 T cells, and other markers, such as CD39, are required to distinguish tumor-specific CD8 T cells [26]. Simoni et al compared tumor-specific CD8 T cells with tumor-unrelated CD8 T cells and found that while PD-1 expression was similar in both tumor-specific and viral-specific CD8 T cells, only the tumor-specific TILs expressed CD39, while the cancer unrelated CD8 TILs did not. This shows that PD-1 is expressed on a broader repertoire of both tumor-reactive and bystander CD8 T cells. Therefore, combining multiple parameters such as PD-1 and 4-1BB may help identify tumor-reactive CD8 TILs that have undergone recent TCR stimulation and gives rationale for using PD-1 and 4-1BB co-expression on CD8 T cells as a potential biomarker to predict response. The emerging importance of composite signatures or profiles by integrating multiple biomarkers is emphasized by recent studies; for instance, CD8 TIL infiltration and physical interaction of PD-1 and PD-L1 expressing cells were associated with clinical response to pembrolizumab in melanoma [27].

A unique feature of INSPIRE is the acquisition of on-treatment tumor biopsies which may identify pharmacodynamic biomarkers that shed insights into mechanisms of biological activity and resistance. The importance of on-treatment biopsies was demonstrated in ICI studies in MM, where CD8 TIL density after pembrolizumab [28] and fold-increase in TIL infiltration after ipilimumab, but not at baseline, was associated with objective responses [29]. Similar to previous studies [28, 29], fold-change in CD8, but not CD4 TILs from baseline positively correlated with response (Fig. 5c-d). On-treatment biopsies were performed coincidentally with the time that most patients had experienced PD-1 blockade in the tumor (Fig. 3). Given that MIH4 and EH12 clones both compete for the same epitope [30], we may consider using a secondary anti-IgG4 antibody in future studies to evaluate the level of PD-1 bound by pembrolizumab to test for occupancy of PD-1 and T cell co-signaling molecule expression kinetics. It may also be relevant to see if clinical response to pembrolizumab or other pharmacodynamic immunologic changes correlate with the degree or kinetics of intratumoral PD-1 blockade.

The current paper aims to share initial results with the community and provide an optimized sample workflow to others who may wish to conduct similar multidisciplinary studies on sequential fresh tumor biopsies. This interim report summarizes the data available from the first 80 enrolled patients, while on-treatment or end-of-treatment samples remain to be collected on these and additional patients at the time of publication. Preliminary translational data such as baseline frequency of 4-1BB+ PD-1+ T cells and intratumoral T cell fold-expansion will be validated with additional techniques and on more patients when data are available. This study has several limitations. Very few progression biopsy samples were collected and have not been analyzed, the study has a short duration of follow-up (4.1 months) and thus survival outcomes are not yet mature, and it is not powered to test for predictive genomic or immune biomarkers in multivariable models. As the number of patients with each tumor type is limited, the correlation between some biomarkers (e.g. PD-1+ 4-1BB+ CD8 T cells and TMB in MM) and the lack of correlation between others (e.g. PD-L1 and TMB) should be considered exploratory requiring validation in larger cohorts. An intriguing parameter that was omitted in this study was the microbiome, which may correlate with anti-tumor immunity and response to ICIs [31, 32]. A study such as this entails an extensive logistical and scientific undertaking that brings together clinicians and multidisciplinary laboratory researchers with the shared goal of understanding the complexities of immunotherapy.

## Additional files

Additional file 1: Supplemental Methods. (DOCX 30 kb)

Additional file 2: **Table S1.** INSPIRE patient baseline characteristics. **Table S2** Optimized flow cytometry panels for INSPIRE. **Table S3** Status of patients as of the June 1, 2017 data cut-off. **Table S4** Adverse events by cohort. **Table S5** c index, *p* values, and false discovery rates (FDRs) for all parameters that were presented in Figs. 4 and 5. (DOCX 52 kb)

Additional file 3: **Figure S1.** INSPIRE trial schema. Pembrolizumab was infused over three-week cycles. Fresh tissue biopsies were collected at baseline (day − 10 to day 0), on-treatment (cycle 2 or 3) at then at the time of tumor progression for the patients who had prolonged stable disease or a partial or complete response. Peripheral blood samples were collected at baseline (S, screening), cycle 1, 2, 3, 5 and every third cycle thereafter and at the end-of-treatment. Follow-up bloods were collected every 12 weeks when feasible. (JPG 1014 kb)

Additional file 4: **Figure S2.** Tumor measurements and time to progression by cohort. Waterfall plots of best percent change in the sum of target tumor lesions (top) and associated time to progression (months; bottom). (JPG 803 kb)

Additional file 5: **Figure S3.** Paired measurements of select immune markers on CD4 and CD8 T cells between fresh tumor biopsies and peripheral blood samples taken at baseline. The median fluorescence intensity of PD-1 (top), 4-1BB (middle), and TIGIT (bottom) were assessed by flow cytometry on CD8 (left) and CD4 (right) T cells from 39 patients who had evaluable flow cytometry data from baseline tumor samples. * *P* < 0.05, ** *P* < 0.01, *** *P* < 0.001. (JPG 1190 kb)

Additional file 6: **Figure S4.** T cell PD-1 occupancy in the peripheral blood and *PDCD1* transcript levels from fresh tumor biopsies at baseline and cycle 2 or 3 of pembrolizumab treatment. Peripheral blood CD8 (left) and CD4 (right) T cell PD-1 occupancy at baseline and weeks three and six post-treatment with pembrolizumab (A). *PDCD1* (*PD1*) transcript abundance in tumor is unchanged after six weeks (two cycles; left) and nine weeks (three cycles; right) of pembrolizumab treatment (B). Paired tumor biopsies are connected by line. (JPG 867 kb)

Additional file 7: **Figure. S5** Pearson correlations between biologically relevant candidate biomarkers. Correlation of TMB and tumor PD-1+ 4-1BB+ CD8 T cells at baseline (A) in all patients, (B) in MM patients, (C) in all patients except MM; PD-L1 MPS and tumor PD-1+ 4-1BB+ CD8 T cells at baseline (D) in all patients, (E) in MM patients, (F) in all patients except MM; TMB and PD-L1 MPS at baseline (G) in all patients, (H) in MM patients, (I) in all patients except MM; and tumor PD-1+ 4-1BB+ CD8 T cells at baseline with fold-expansion of (J) tumor CD8 T cells and (K) tumor CD4 T cells. Orange, SCCHN; Pink, TNBC; Purple, HGSC; Red, MM; Blue, MST. (JPG 446 kb)

Additional file 8: **Figure S6.** Effect of filtering and tumour type on assessing TMB by exome sequencing. (A) Comparison of total number of non-synonymous mutations detected in each pre-treatment tumour sample (*N* = 50) with or without minimum variant allele fraction (VAF) threshold applied (VAF > 10%). Pearson correlation and raw *p*-value for correlation test are show. (B) Boxplot comparison of pre-treatment TMB (total number of non-synonymous mutations) between clinical response groups by RECIST1.1 with or without VAF filter. Statistical significance evaluated using one-way ANOVA. (C) Boxplot comparison of pre-treatment TMB between disease cohorts. Statistically significant differences in TMB between cohorts A to D was evaluated using one-way ANOVA. Cohort E (mixed solid tumor cohort) was excluded from this comparison Additional file 8: Figure S6. (JPG 238 kb)

Additional file 9: **Table S6.** Exome sequencing data quality data from baseline samples using Picard Tools v.2.6.0 Additional file 9: Table S6. (XLSX 76 kb)

## Abbreviations

AE: Adverse event
CN: Copy number
CR: Complete response
FDR: False discovery rate
FFPE: Formalin-fixed paraffin-embedded
HGSC: High-grade serous ovarian cancer
ICI: Immune checkpoint inhibitor
IHC: Immunohistochemistry
INSPIRE: Investigator initiated Phase 2 study of pembrolizumab immunological response evaluation
MDSC: Myeloid derived suppressor cells
MFI: Median fluorescence intensity
MM: Metastatic melanoma
MPS: Modified proportion score
MST: Mixed advanced solid tumors
PCR: Polymerase chain reaction
PD: Progressive disease
PD-1: Programmed cell death 1
PD-L1: Programmed cell death 1 ligand
PGA: Percent genome altered
PM-OICR TGL: Princess Margaret – Ontario Institute of Cancer Research Translational Genomics Laboratory
PR: Partial response
RECIST1.1: Response evaluation criteria in solid tumors version 1.1
SCCHN: Squamous cell carcinoma of the head and neck
SNV: Single nucleotide variant
TCR: T cell receptor
TIL: Tumor infiltrating lymphocyte
TMB: Tumor mutation burden
TNBC: Triple negative breast cancer
UTR: Untranslated region
WES: Whole exome sequencing
