# Supplementary material for: An interim report on the investigator-initiated phase 2 study of pembrolizumab immunological response evaluation (INSPIRE)
Source: J Immunother Cancer. 2019 Mar 13;7:72. doi: 10.1186/s40425-019-0541-0 (PMC6417194; doi:10.1186/s40425-019-0541-0)
Supplement: Supplementary file 1 — Supplemental Methods. (DOCX 30 kb) [file 40425_2019_541_MOESM1_ESM.docx]

**Supplemental Methods**

**Patient selection and sample collection.** Inclusion criteria included Eastern Cooperative Group (ECOG) performance status 0 or 1; adequate organ function; and willingness to provide fresh tumor tissue for biomarker evaluation. Exclusion criteria included prior therapy with any anti-PD1/L1/L2 antibodies (prior anti-cytotoxic T-lymphocyte-associated antigen 4 (CTLA4) and T cell co-stimulatory agents were allowed); symptomatic or unstable brain metastases; known active hepatitis B or C, or human immunodeficiency virus infection; active autoimmune disease requiring systemic treatment in past two years; pregnant or lactating females; history or any evidence of active, non-infectious pneumonitis; and other serious medical disorders. Patients required a washout period of at least four weeks for prior anticancer therapies before starting study treatment. Patients were considered evaluable if they had fresh tumor samples available from the baseline and on-treatment biopsies, with acceptable quality and quantity for correlative analysis. Peripheral blood samples were taken at baseline, cycle one (C1), C2, C3, C5, every third cycle thereafter, and at the end of treatment for flow cytometry; and at baseline, C1, C3, every third cycle thereafter, and at the end of treatment for ctDNA.

**Drug administration.** Dose interruptions (up to 21 days) were permitted in the event of study drug-related toxicity. Subjects continued to receive study treatment until they experienced disease progression, unacceptable adverse events, or withdrawal of consent. Patients could complete 24 months of uninterrupted treatment with pembrolizumab or 35 administrations, whichever is later. Patients with confirmed radiological progression by Response Evaluation Criteria in Solid Tumors (RECIST) version 1.1 were allowed to continue on treatment with pembrolizumab if clinically stable or clinically improved.

**Safety and efficacy assessment**. Safety assessments included physical examination, vital signs, weight, performance status assessment and documentation of adverse events and serious adverse events. Laboratory tests including hematology, coagulation, biochemistry, urinalysis, pregnancy test, if applicable, and thyroid function tests were performed during screening, on-treatment and end of treatment at protocol specified time points and at any time when clinically indicated. Tumor assessments by CT or MRI were performed at baseline and approximately every nine weeks during treatment, and at the end of treatment. All subjects who received at least one dose of pembrolizumab were evaluated for safety parameters. Additionally, any occurrence of a serious adverse event from the time of consent until 30 days post discontinuation of study drug dosing was documented. Any occurrence of non-serious adverse events was collected from first dose of study drug until 30 days post discontinuation of dosing. Safety was evaluated for all treated subjects using the National Cancer Institute’s Common Toxicity Criteria for Adverse Events version 4.03.

**IHC tissue handling.** The FFPE/IHC core or tissue fragment was placed in a 60mL collection container with 30mL of 10% neutral buffered formalin for 12-24 hours, with a maximum fixation time of 96 hours at room temperature. Remaining core biopsies were stored in normal saline at room temperature before fresh processing (within four hours of collection). For screening biopsies only, the FFPE blocks were used for PD-L1 IHC (clone 22C3) on 4-5 micron sections mounted on positively charged ProbeOn slides (QualTek, Goleta, CA). QualTek provided a modified proportion score (MPS) indicating the proportion of PD-L1-expressing tumor cells and mononuclear inflammatory cells within tumor nests. MPS was calculated as the percent of PD-L1 positive mononuclear inflammatory cells such as T, B, NK, macrophages, dendritic cells plus PD-L1 positive tumor cells within tumor nests. Granulocytes and plasma cells were excluded as they are part of an inflammatory response as opposed to an immune-specific response from lymphocytes (T, B and NK), macrophages and dendritic cells. Granulocytes and plasma cells are not calculated if they are within a tumor nest. Other cell types within a tumor nest could be endothelial (blood/lymph vessels), stromal fibroblasts and occasionally metaplastic components (e.g., osseous metaplasia) and other potentially entrapped non-neoplastic cells (tumor growing through normal). Other non-cellular components such as mucin, collagen, hemosiderin, anthracotic pigment and melanin pigment were also not considered.

**DNA/RNA extraction.** Genomic DNA and RNA were co-extracted from flash-frozen tumor and peripheral-blood mononuclear cell (PBMC) pellets using the Qiagen AllPrep DNA/RNA/miRNA Universal Kit (Toronto, Ontario, Canada). DNA and RNA concentrations were measured using the Qubit fluorometer and double stranded DNA and RNA Broad Range assay kits (Life Technologies, Burlington, Ontario, Canada).

**Data Analysis and Statistics.**

Sequencing library quality of all samples was assessed using Picard Tools v.2.6.0 (**Table S6**). Somatic copy number alteration profiles, tumor cellularity and ploidy estimations were determined using Sequenza v2.1.2 [1] following variant detection from Varscan2 v2.4.2 [2]. Somatic point mutations and small insertions and deletions for each tumor were identified using MuTect2 [3] with default settings and using paired tumor and normal WES data. Variant Effect Predictor v83 [4] was used to annotate the functional consequences of detected somatic sequence mutations. Total number of non-synonymous somatic mutations in each tumor was calculated a count of the total number of non-synonymous somatic sequence variants detected exceeding 10% variant allele frequency. Griffith et al*.* has demonstrated through evaluation of the positive-predictive value of seven commonly used variant callers on 300X whole genome sequencing tumor data that none of the seven callers are able to detect variants with VAF < 10% with high confidence [5]. Mutation burden pre-and post-VAF filtering was highly correlated **(Figure S6A)** and unfiltered data did not change the outcome of the TMB analysis between responders and non-responders **(Figure S6B)** or by tumor cohort (**Figure S6C**). To calculate the percentage of genome altered (PGA) by somatic copy number alterations, first, total absolute copy number is determined for each protein coding gene, then percentage of genes with total copy number greater or less than two is quantified for PGA. Separately, the percentage genome copy number gain was calculated as the percentage of protein coding genes with total copy number greater than two, and percentage genome copy number loss was calculated as the percentage of protein coding genes with total copy number less than two.

Assuming a type I error rate of alpha=0.05 and an effect size of one standard deviation (SD), the entire INSPIRE cohort (assumed *N*=100) was powered at 85, 95, and 98% to detect significant differences given response rates of 10, 15, and 20%, respectively. Within each cohort (assumed *N*=20), assuming an effect size of 1SD, the study was powered at 27, 49, 61% to detect statistically significant differences given response rates of 10, 25, and 50%, respectively.

**Supplemental References**

1. Favero F, Joshi T, Marquard AM et al. Sequenza: allele-specific copy number and mutation profiles from tumor sequencing data. Ann Oncol 2015; 26: 64-70.

2. Koboldt DC, Zhang Q, Larson DE et al. VarScan 2: somatic mutation and copy number alteration discovery in cancer by exome sequencing. Genome Res 2012; 22: 568-576.

3. McKenna A, Hanna M, Banks E et al. The Genome Analysis Toolkit: a MapReduce framework for analyzing next-generation DNA sequencing data. Genome Res 2010; 20: 1297-1303.

4. McLaren W, Gil L, Hunt SE et al. The Ensembl Variant Effect Predictor. Genome Biol 2016; 17: 122.

5. Griffith M, Miller CA, Griffith OL et al. Optimizing cancer genome sequencing and analysis. Cell Syst 2015; 1: 210-223.
