# Supplementary material for: An interim report on the investigator-initiated phase 2 study of pembrolizumab immunological response evaluation (INSPIRE)
Source: J Immunother Cancer. 2019 Mar 13;7:72. doi: 10.1186/s40425-019-0541-0 (PMC6417194; doi:10.1186/s40425-019-0541-0)
Supplement: Supplementary file 2 — Table S1. INSPIRE patient baseline characteristics. Table S2 Optimized flow cytometry panels for INSPIRE. Table S3 Status of patients as of the June 1, 2017 data cut-off. Table S4 Adverse events by cohort. Table S5 c index, p values, and false discovery rates (FDRs) for all parameters that were presented in Figs. 4 and 5. (DOCX 52 kb) [file 40425_2019_541_MOESM2_ESM.docx]

**Table S1** INSPIRE patient baseline characteristics.

| **Characteristic** |  |
| --- | --- |
| **Age, median (range)** | 58 (21-82) |
| **Gender, *N* (%)**  male  female | 28 (35)  52 (65) |
| **Diagnosis, *N* (%)**  SCCHN  TBNC  HGSC  MM*  MST** | 8 (10)  13 (16)  21 (26)  10 (12)  28 (35) |
| **ECOG PS, *N* (%)**  0  1 | 24 (30)  56 (70) |
| **Adjuvant therapy, *N* (%)**  yes  no | 42 (53)  38 (47) |
| **Types and prior lines of systemic therapy^§^, *N* (%)**  0  1  2  3+  median (range)  chemotherapy  targeted therapy  immunotherapy – anti-CTLA4  immunotherapy – anti-PD1/L1  immunotherapy – other | 17 (21)  26 (33)  17 (21)  20 (25)  1 (0-7)  51 (64)  27 (34)  4 (5)  0 (0)  3 (4) |
| **Number of metastatic sites, *N* (%)**  0  1  2  3+ | 0 (0)  6 (8)  16 (20)  58 (72) |
| **Baseline PD-L1 score by MPS, *N* (%)**  0  1-10  11-25  26-49  50+  Not evaluable or missing | 41 (51)  19 (24)  6 (8)  3 (4)  8 (10)  3 (4) |

* MM = cutaneous (7), choroidal (1), desmoplastic (1), vaginal (1).

** MST = Merkel cell carcinoma (3), adenoid cystic cancer (2), endometrial cancer (2), MSI colorectal cancer (2), nasopharyngeal cancer (2), solitary fibrous tumor (2), alveolar soft part sarcoma (1), anal canal small cell cancer (1), anal canal squamous cell cancer (1), basal cell cancer with squamous differentiation (1), cervix cancer (1), cholangiocarcinoma (1), chondrosarcoma (1), clear cell sarcoma (1), endometrial clear cell carcinoma (1), gastroesophageal junction adenocarcinoma (1), granular cell tumor (1) , myxoid liposarcoma (1), neuroendocrine tumor of cervix (1), penile squamous cell cancer (1), spindle cell cancer of head and neck (1).

§ for recurrent or metastatic disease only; not including adjuvant.

**Table S2** Optimized flow cytometry panels for INSPIRE.

| **Epitope** | **Fluor** | **Company** | **Cat. No.** | **Dilution** |
| --- | --- | --- | --- | --- |
| **T cell activation/exhaustion markers** | | |  |  |
| TCRgd | **FITC** | eBioscience | 11-9959-42 | 1:50 |
| CD8 | **PerCP** | BioLegend | 301030 | 1:50 |
| CD137 | **PE** | eBioscience | 12-1379-42 | 1:100 |
| TIGIT | **PE-Cy7** | eBioscience | 25-9500-42 | 1:50 |
| CTLA-4 (surface) | **eFluor660** | eBioscience | 50-1529-42 | 1:100 |
| CD4 | **Alexa700** | eBioscience | 56-0048-42 | 1:100 |
| CD19 | **Alexa700** | eBioscience | 56-0199-42 | 1:100 |
| CD56 | **APC-Cy7** | BioLegend | 318332 | 1:100 |
| PD-L1 | **eF450** | eBioscience | 48-5983-42 | 1:50 |
| fixable viability | **e506** | eBioscience | 65-0866-18 | 1:800 |
| PD-1 | **BV605** | BioLegend | 329924 | 1:100 |
| CD3 | **BUV395** | BD | 563546 | 1:25 |
|  |  |  |  |  |
| **Treg panel** |  |  |  |  |
| Ki67 | **FITC** | eBioscience | 11-5699-42 | 1:50 |
| CD39 | **PerCP-eF710** | eBioscience | 46-0399-42 | 1:100 |
| FOXP3 | **PE** | eBioscience | 12-0399-42 | 1:20 |
| CTLA-4 (intracellular) | **PE-Cy7** | eBioscience | 25-1529-42 | 1:100 |
| CD25 | **APC** | eBioscience | 17-0259-42 | 1:25 |
| CD8 | **Alexa700** | eBioscience | 56-0088-42 | 1:100 |
| Helios | **APC-eF780** | eBioscience | 47-9883-42 | 1:20 |
| CD127 | **eF450** | eBioscience | 48-1278-42 | 1:100 |
| fixable viability | **e506** | eBioscience | 65-0866-42 | 1:800 |
| CD4 | **BV605** | BD | 562658 | 1:50 |
| CD3 | **BUV395** | BD | 563546 | 1:25 |
|  |  |  |  |  |
| **T/B/NK panel** |  |  |  |  |
| LAG3 | **FITC** | eBioscience | 11-2239-42 | 1:100 |
| CD8 | **PerCP** | BioLegend | 301030 | 1:100 |
| CD103 | **PE** | eBioscience | 12-1038-42 | 1:100 |
| CD28 | **PE-Cy7** | eBioscience | 25-0289-42 | 1:100 |
| TIM3 | **APC** | R&D | FAB2365A | 1:50 |
| CD4 | **Alexa700** | eBioscience | 56-0048-42 | 1:100 |
| CD19 | **Alexa700** | eBioscience | 56-0199-42 | 1:100 |
| CD56 | **APC-Cy7** | BioLegend | 318332 | 1:100 |
| HLA-DR | **eF450** | eBioscience | 48-9956-42 | 1:100 |
| fixable viability | **e506** | eBioscience | 65-0866-18 | 1:800 |
| PD-1 | **BV605** | BioLegend | 329924 | 1:100 |
| CD3 | **BUV395** | BD | 563546 | 1:25 |

**Table S3** Status of patients as of the June 1, 2017 data cut-off.

|  | **Cohort A**  **SCCHN** | **Cohort B**  **TNBC** | **Cohort C**  **HGSC** | **Cohort D**  **MM** | **Cohort E**  **MST** |
| --- | --- | --- | --- | --- | --- |
| **Treated, *N*** | **8** | **13** | **21** | **10** | **28** |
| **Median duration, *N* (range)** | | | | | |
| weeks | 9 (3-48) | 6 (2-15) | 9 (0-38) | 25 (3-57) | 15 (0-47) |
| number of cycles | 4 (2-17) | 3 (1-5) | 3 (1-12) | 10 (1-20) | 5 (1-15) |
| continuing treatment | 4 (50) | 3 (23) | 2 (10) | 7 (70) | 7 (25) |
| discontinued treatment | 4 (50) | 10 (77) | 19 (90) | 3 (30) | 21 (75) |
| **Reason for discontinuation, *N* (%)** | | | | | |
| AEs, side effects, complications | 0 (0) | 0 (0) | 4 (21) | 0 (0) | 0 (0) |
| alternative therapy | 0 (0) | 0 (0) | 0 (0) | 0 (0) | 1 (5) |
| PD, relapse during treatment | 4 (100) | 10 (100) | 15 (79) | 3 (100) | 18 (86) |
| other* | 0 (0) | 0 (0) | 0 (0) | 0 (0) | 1 (5) |
| patient withdrawal or refusal | 0 (0) | 0 (0) | 0 (0) | 0 (0) | 1 (5) |
| **Best objective response, *N* (%)** | | | | | |
| CR | 0 (0) | 0 (0) | 0 (0) | 0 (0) | 0 (0) |
| PR | 2 (25) | 1 (8) | 0 (0) | 6 (60) | 4 (15) |
| SD | 3 (38) | 3 (23) | 6 (29) | 0 (0) | 9 (33) |
| PD | 3 (38) | 9 (69) | 14 (67) | 3 (30) | 14 (52) |
| not evaluable or missing* | 0 (0) | 0 (0) | 1 (5) | 1 (10) | 1 (5)** |
| follow-up time, months | 0.7-10.6 | 1.1-9 | 0-12.4 | 0.7-11.7 | 0.7-13.8 |
| progressed | 4 (50) | 10 (77) | 16 (76) | 3 (30) | 18 (64) |
| TTP, median (95% CI) | 3.4  (1.4-NE) | 2.0  (1-3.5) | 2.8  (1.9-3.9) | NE | 3.5  (3.4-7.9) |

* patient INS-E-028 was taken off study due to intercurrent tuberculosis infection and abdominal abscess.
** data missing because one patient withdrew consent**.**

AE, adverse event; NE, not estimable; TTP, time to progression

**Table S4** Adverse events by cohort.

| AE term (Grade 1-2); **cohort:** histology | **A:** SCCHN | **B:** TNBC | **C:** HGSC | **D:** MM | **E:** MST | total |
| --- | --- | --- | --- | --- | --- | --- |
| ABDOMINAL PAIN | 0 (0) | 1 (8) | 2 (10) | 0 (0) | 0 (0) | 3 (4) |
| ALANINE AMINOTRANSFERASE INCREASED | 0 (0) | 0 (0) | 4 (19) | 0 (0) | 0 (0) | 4 (5) |
| ALKALINE PHOSPHATASE INCREASED | 0 (0) | 0 (0) | 4 (19) | 0 (0) | 0 (0) | 4 (5) |
| ANEMIA | 0 (0) | 1 (8) | 3 (14) | 0 (0) | 0 (0) | 4 (5) |
| ANOREXIA | 0 (0) | 2 (15) | 2 (10) | 0 (0) | 0 (0) | 4 (5) |
| ARTHRALGIA | 0 (0) | 0 (0) | 3 (14) | 3 (30) | 5 (18) | 11 (14) |
| ASPARTATE AMINOTRANSFERASE INCREASED | 1 (13) | 0 (0) | 5 (24) | 0 (0) | 0 (0) | 6 (8) |
| AUTOIMMUNE DISORDER | 0 (0) | 0 (0) | 0 (0) | 0 (0) | 1 (4) | 1 (1) |
| BACK PAIN | 0 (0) | 0 (0) | 1 (5) | 0 (0) | 0 (0) | 1 (1) |
| BLURRED VISION | 0 (0) | 0 (0) | 1 (5) | 0 (0) | 0 (0) | 1 (1) |
| CHILLS | 0 (0) | 1 (8) | 1 (5) | 0 (0) | 2 (7) | 4 (5) |
| COUGH | 0 (0) | 0 (0) | 2 (10) | 2 (20) | 0 (0) | 4 (5) |
| DEHYDRATION | 0 (0) | 0 (0) | 2 (10) | 0 (0) | 1 (4) | 3 (4) |
| DEPRESSION | 1 (13) | 0 (0) | 1 (5) | 0 (0) | 0 (0) | 2 (3) |
| DIARRHEA | 1 (13) | 2 (15) | 7 (33) | 2 (20) | 5 (18) | 17 (21) |
| DIZZINESS | 0 (0) | 0 (0) | 0 (0) | 0 (0) | 1 (4) | 1 (1) |
| DRY MOUTH | 0 (0) | 1 (8) | 1 (5) | 0 (0) | 0 (0) | 2 (3) |
| DRY SKIN | 0 (0) | 1 (8) | 0 (0) | 0 (0) | 1 (4) | 2 (3) |
| DYSPEPSIA | 0 (0) | 0 (0) | 4 (19) | 0 (0) | 0 (0) | 4 (5) |
| DYSPHAGIA | 1 (13) | 0 (0) | 0 (0) | 0 (0) | 0 (0) | 1 (1) |
| DYSPNEA | 0 (0) | 0 (0) | 3 (14) | 2 (20) | 3 (11) | 8 ( 0) |
| EDEMA LIMBS | 0 (0) | 0 (0) | 1 (5) | 0 (0) | 0 (0) | 1 (1) |
| ERYTHEMA MULTIFORME | 1 (13) | 0 (0) | 0 (0) | 0 (0) | 0 (0) | 1 (1) |
| FATIGUE | 2 (25) | 3 (23) | 12 (57) | 3 (30) | 10 (36) | 30 (38) |
| FEVER | 0 (0) | 0 (0) | 2 (10) | 1 (10) | 1 (4) | 4 (5) |
| FLU LIKE SYMPTOMS | 0 (0) | 0 (0) | 2 (10) | 0 (0) | 0 (0) | 2 (3) |
| GASTROESOPHAGEAL REFLUX DISEASE | 0 (0) | 0 (0) | 0 (0) | 0 (0) | 1 (4) | 1 (1) |
| GLUCOSE INTOLERANCE | 0 (0) | 0 (0) | 2 (0) | 0 (0) | 0 (0) | 2 (3) |
| HEADACHE | 0 (0) | 0 (0) | 3 (4) | 1 (10) | 1 (4) | 5 (6) |
| HYPERHIDROSIS | 0 (0) | 0 (0) | 0 (0) | 0 (0) | 1 (4) | 1 (1) |
| HYPERKALEMIA | 0 (0) | 1 (8) | 0 (0) | 0 (0) | 0 (0) | 1 (1) |
| HYPERTENSION | 0 (0) | 0 (0) | 1 (5) | 0 (0) | 0 (0) | 1 (1) |
| HYPERTHYROIDISM | 0 (0) | 0 (0) | 1 (5) | 2 (20) | 0 (0) | 3 (4) |
| HYPOALBUMINEMIA | 0 (0) | 0 (0) | 1 (5) | 0 (0) | 0 (0) | 1 (1) |
| HYPOMAGNESEMIA | 0 (0) | 0 (0) | 1 (5) | 0 (0) | 2 (7) | 3 (4) |
| HYPONATREMIA | 0 (0) | 0 (0) | 3 (14) | 0 (0) | 0 (0) | 3 (4) |
| HYPOPHOSPHATEMIA | 0 (0) | 0 (0) | 0 (0) | 1 (10) | 0 (0) | 1 (1) |
| HYPOTENSION | 0 (0) | 0 (0) | 0 (0) | 0 (0) | 1 (4) | 1 (1) |
| HYPOTHYROIDISM | 1 (13) | 0 (0) | 1 (5) | 3 (30) | 2 (7) | 7 (9) |
| INSOMNIA | 0 (0) | 0 (0) | 1 (5) | 1 (10) | 0 (0) | 2 (3) |
| LIPASE INCREASED | 0 (0) | 0 (0) | 2 (10) | 0 (0) | 0 (0) | 2 (3) |
| MALAISE | 0 (0) | 0 (0) | 0 (0) | 0 (0) | 1 (4) | 1 (1) |
| MEMORY IMPAIRMENT | 0 (0) | 0 (0) | 1 (5) | 0 (0) | 0 (0) | 1 (1) |
| MYALGIA | 0 (0) | 0 (0) | 3 (14) | 2 (20) | 2 (7) | 7 (9) |
| NAUSEA | 0 (0) | 1 (8) | 7 (33) | 0 (0) | 5 (18) | 13 (16) |
| NON-CARDIAC CHEST PAIN | 0 (0) | 0 (0) | 1 (5) | 0 (0) | 0 (0) | 1 (1) |
| OCULOMOTOR NERVE DISORDER | 0 (0) | 0 (0) | 1 (5) | 0 (0) | 0 (0) | 1 (1) |
| OTHER | 1 (13) | 1 (8) | 8 (38) | 6 (60) | 3 (11) | 19 (24) |
| PERIPHERAL SENSORY NEUROPATHY | 0 (0) | 0 (0) | 1 (5) | 0 (0) | 0 (0) | 1 (1) |
| PLATELET COUNT DECREASED | 1 (13) | 0 (0) | 2 (10) | 0 (0) | 0 (0) | 3 (4) |
| PNEUMONITIS | 0 (0) | 0 (0) | 0 (0) | 1 (10) | 0 (0) | 1 (1) |
| PROCTITIS | 0 (0) | 0 (0) | 1 (5) | 0 (0) | 0 (0) | 1 (1) |
| PROTEINURIA | 0 (0) | 0 (0) | 1 (5) | 0 (0) | 0 (0) | 1 (1) |
| PRURITUS | 1 (13) | 1 (8) | 3 (14) | 2 (20) | 4 (14) | 11 (14) |
| RASH ACNEIFORM | 1 (13) | 0 (0) | 1 (5) | 1 (10) | 3 (11) | 6 (8) |
| RASH MACULO-PAPULAR | 2 (25) | 1 (8) | 0 (0) | 1 (10) | 3 (11) | 7 (9) |
| RESTLESSNESS | 0 (0) | 0 (0) | 1 (5) | 0 (0) | 0 (0) | 1 (1) |
| SERUM AMYLASE INCREASED | 0 (0) | 0 (0) | 1 (5) | 0 (0) | 0 (0) | 1 (1) |
| UPPER RESPIRATORY INFECTION | 0 (0) | 0 (0) | 0 (0) | 1 (10) | 0 (0) | 1 (1) |
| URINARY TRACT INFECTION | 0 (0) | 0 (0) | 1 (5) | 0 (0) | 0 (0) | 1 (1) |
| VOMITING | 0 (0) | 2 (15) | 2 (10) | 0 (0) | 0 (0) | 4 (5) |
| AE term (Grade 3-4); **cohort:** histology | **A:** SCCHN | **B:** TNBC | **C:** HGSC | **D:** MM | **E:** MST | total |
| ABDOMINAL INFECTION | 0 (0) | 0 (0) | 0 (0) | 0 (0) | 1 (4) | 1 (1) |
| DIARRHEA | 0 (0) | 0 (0) | 1 (5) | 0 (0) | 0 (0) | 1 (1) |
| FATIGUE | 0 (0) | 0 (0) | 2 (10) | 0 (0) | 0 (0) | 2 (3) |
| HYPERTENSION | 0 (0) | 0 (0) | 1 (5) | 0 (0) | 0 (0) | 1 (1) |
| HYPONATREMIA | 0 (0) | 0 (0) | 1 (5) | 0 (0) | 0 (0) | 1 (1) |
| LIPASE INCREASED | 0 (0) | 0 (0) | 1 (5) | 0 (0) | 0 (0) | 1 (1) |
| OTHER | 0 (0) | 0 (0) | 1 (5) | 0 (0) | 0 (0) | 1 (1) |
| PNEUMONITIS | 0 (0) | 1 (8) | 0 (0) | 1 (10) | 0 (0) | 2 (3) |
| PROTEINURIA | 0 (0) | 0 (0) | 1 (5) | 0 (0) | 0 (0) | 1 (1) |
| RECTAL FISTULA | 0 (0) | 0 (0) | 1 (5) | 0 (0) | 0 (0) | 1 (1) |
| SERUM AMYLASE INCREASED | 0 (0) | 0 (0) | 1 (5) | 0 (0) | 0 (0) | 1 (1) |

**Table S5** c index, p values, and false discovery rates (FDRs) for all parameters that were presented in Figs. 4 and 5.

| **all listed as:**  **c index**  **p value**  **FDR** | **without stratification**  **by tumor type** | **with stratification**  **by tumor type** |
| --- | --- | --- |
| baseline tumor  total PGA | 0.27  1.5E-07  6.06E-06 | 0.34  6.5E-03  7.26E-02 |
| baseline tumor  CN gain | 0.33  5.7E-04  1.15E-02 | 0.38  2.6E-02  1.58E-01 |
| baseline tumor  CN loss | 0.27  8.7E-07  2.61E-05 | 0.38  8.2E-02  3.48E-01 |
| baseline tumor  gd T cells | 0.29  3.5E-03  3.47E-02 | 0.32  1.5E-02  1.03E-01 |
| baseline tumor  PD1 41BB CD8 T cell | 0.71  5.8E-04  1.15E-02 | 0.71  2.8E-03  3.55E-02 |
| on tx CD4 T cell fold change (tumor) | 0.68  5.1E-02  2.77E-01 | 0.76  2.8E-02  1.61E-01 |
| on tx CD8 T cell fold change (tumor) | 0.73  4.3E-03  3.98E-02 | 0.66  1.3E-01  4.29E-01 |
| on tx CD4 T cell fold change at C3 (blood) | 0.63  7.6E-03  6.54E-02 | 0.54  4.9E-01  7.79E-01 |
