# Supplementary figures and images for: An interim report on the investigator-initiated phase 2 study of pembrolizumab immunological response evaluation (INSPIRE)
Source: J Immunother Cancer. 2019 Mar 13;7:72. doi: 10.1186/s40425-019-0541-0 (PMC6417194; doi:10.1186/s40425-019-0541-0)

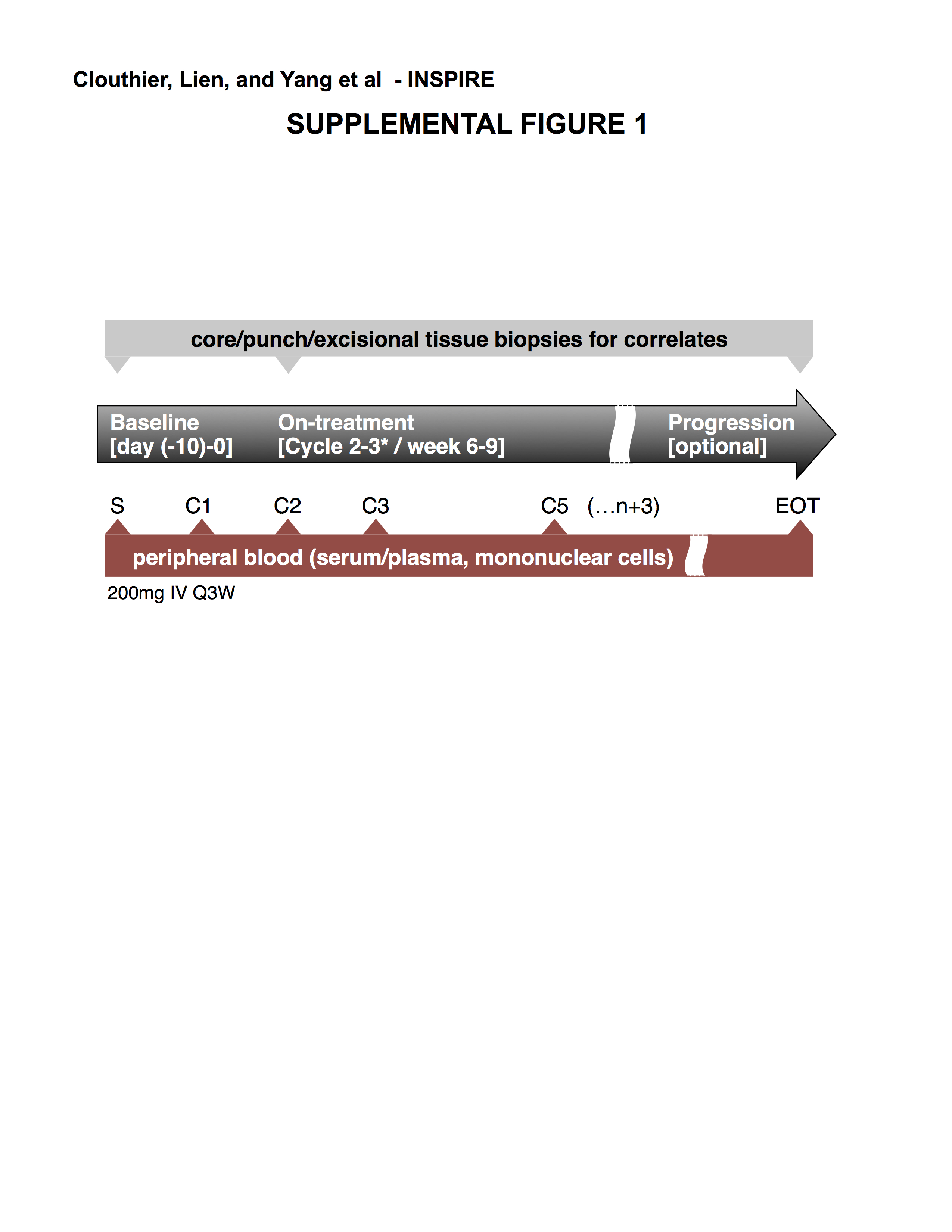

Supplement: Supplementary file 3 — Figure S1. INSPIRE trial schema. Pembrolizumab was infused over three-week cycles. Fresh tissue biopsies were collected at baseline (day − 10 to day 0), on-treatment (cycle 2 or 3) at then at the time of tumor progression for the patients who had prolonged stable disease or a partial or complete response. Peripheral blood samples were collected at baseline (S, screening), cycle 1, 2, 3, 5 and every third cycle thereafter and at the end-of-treatment. Follow-up bloods were collected every 12 weeks when feasible. (JPG 1014 kb) [file 40425_2019_541_MOESM3_ESM.jpg]

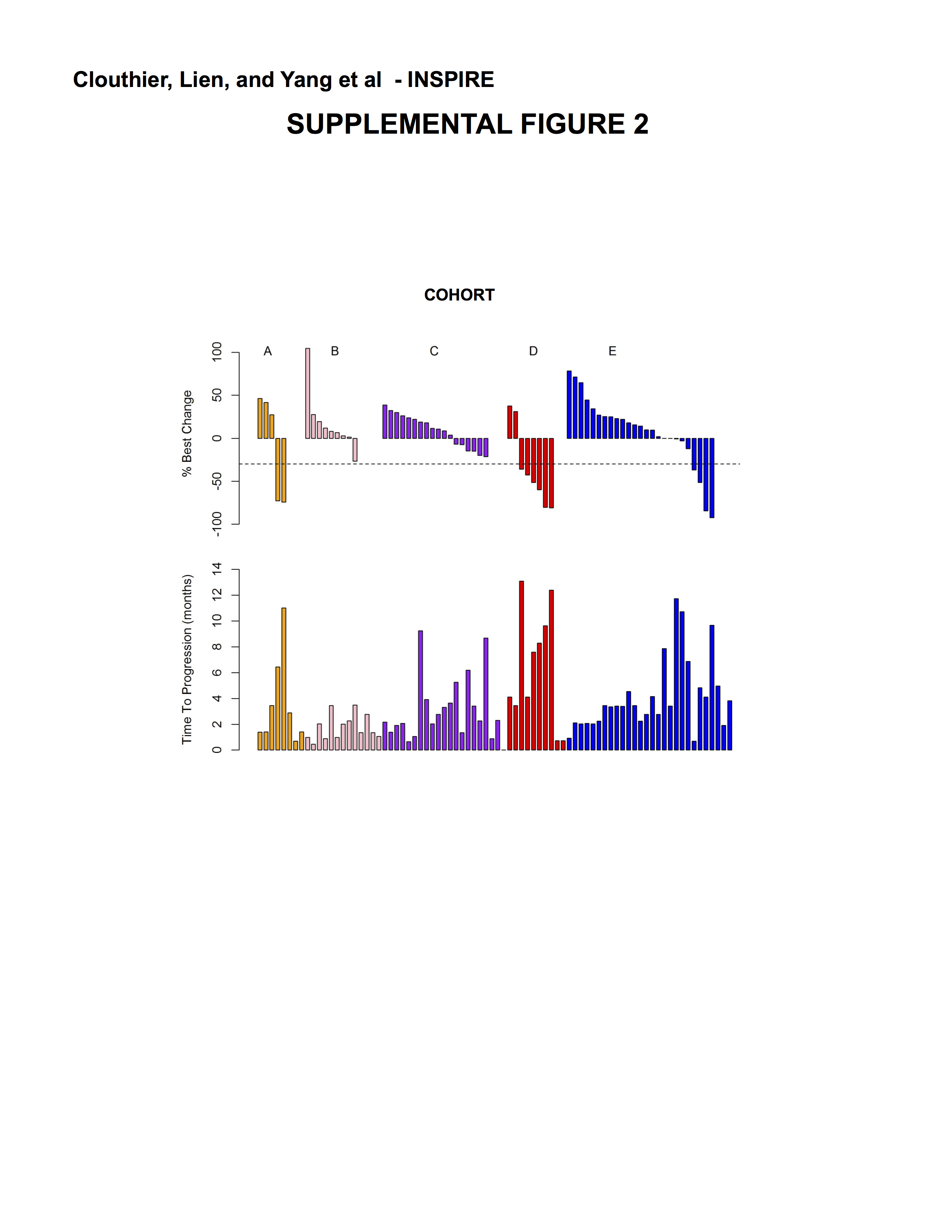

Supplement: Supplementary file 4 — Figure S2. Tumor measurements and time to progression by cohort. Waterfall plots of best percent change in the sum of target tumor lesions (top) and associated time to progression (months; bottom). (JPG 803 kb) [file 40425_2019_541_MOESM4_ESM.jpg]

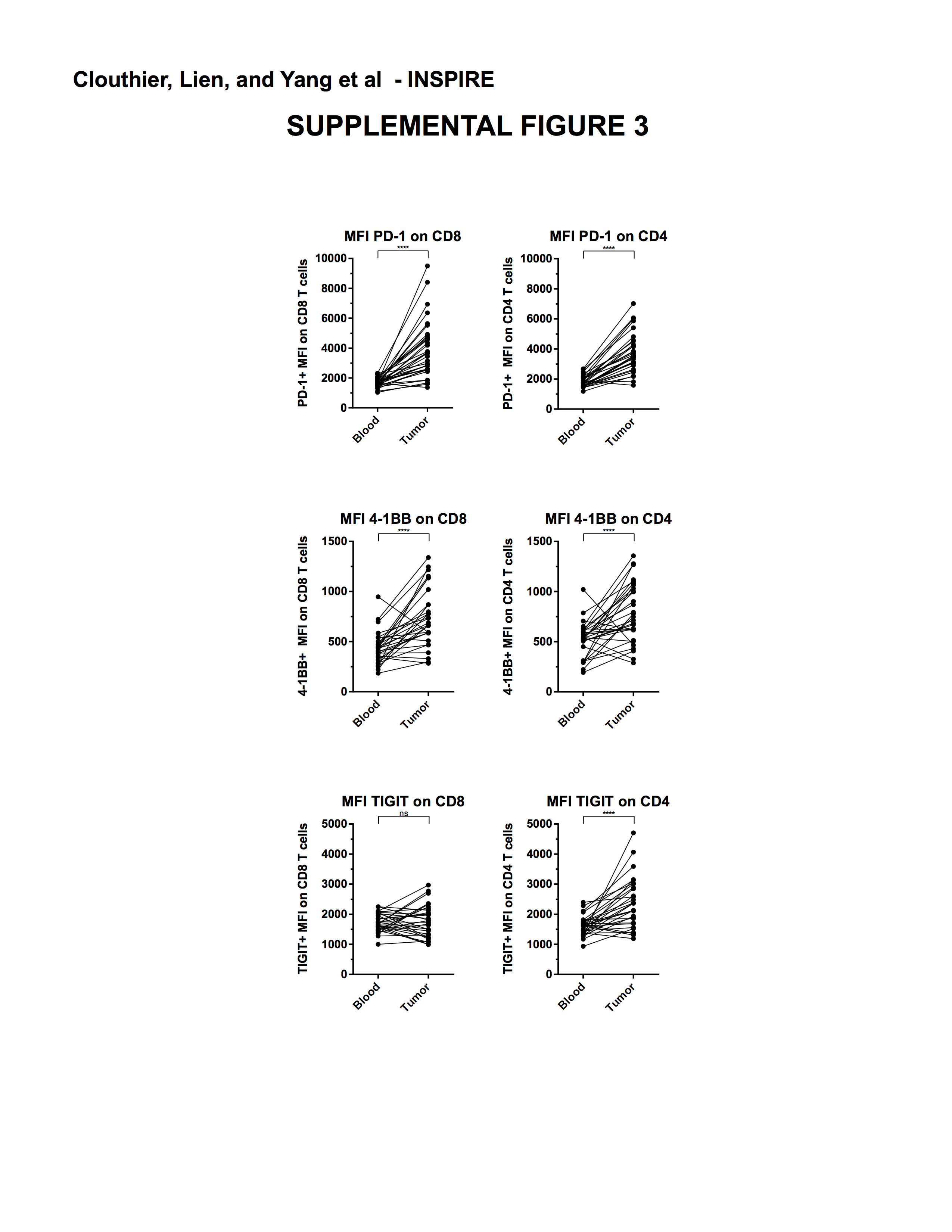

Supplement: Supplementary file 5 — Figure S3. Paired measurements of select immune markers on CD4 and CD8 T cells between fresh tumor biopsies and peripheral blood samples taken at baseline. The median fluorescence intensity of PD-1 (top), 4-1BB (middle), and TIGIT (bottom) were assessed by flow cytometry on CD8 (left) and CD4 (right) T cells from 39 patients who had evaluable flow cytometry data from baseline tumor samples. * P < 0.05, ** P < 0.01, *** P < 0.001. (JPG 1190 kb) [file 40425_2019_541_MOESM5_ESM.jpg]

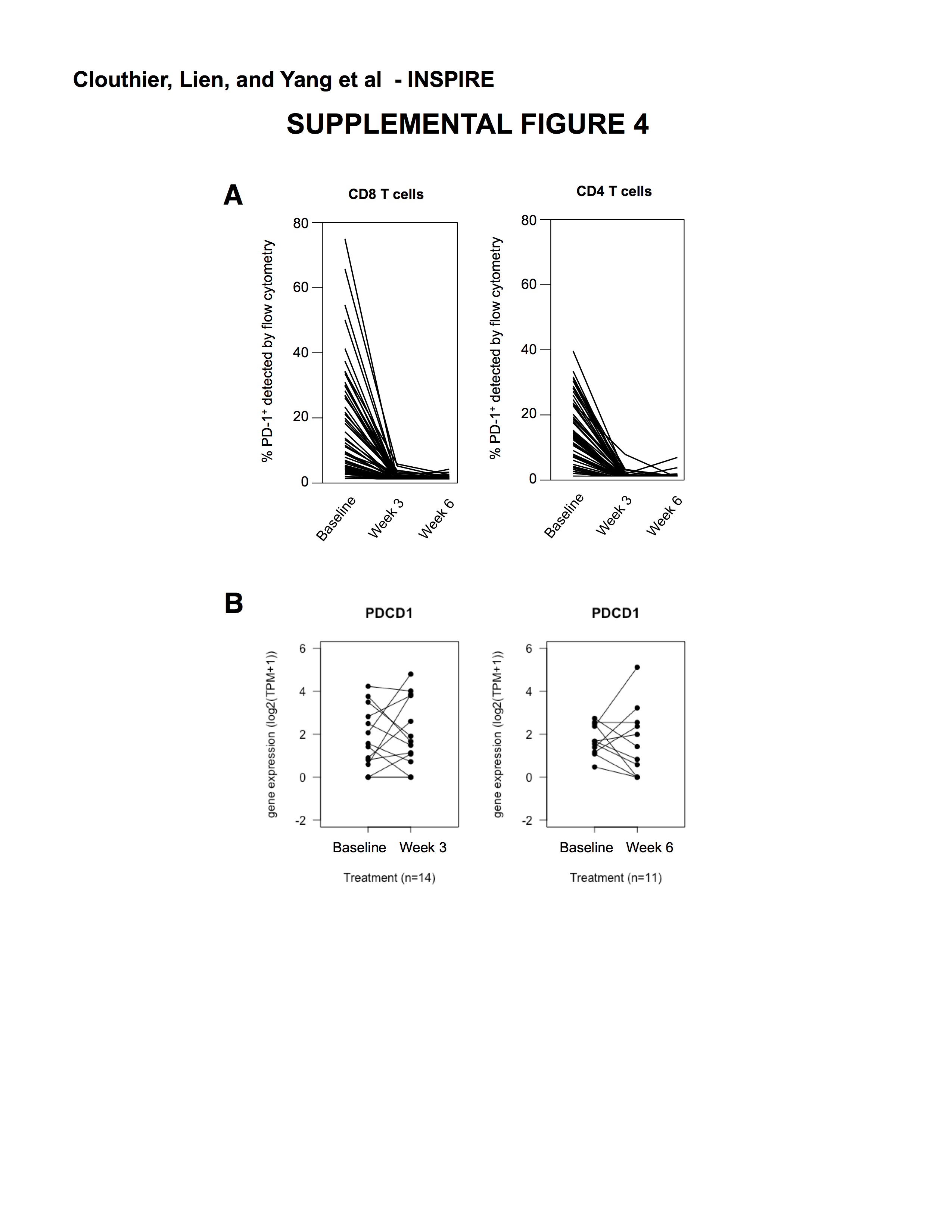

Supplement: Supplementary file 6 — Figure S4. T cell PD-1 occupancy in the peripheral blood and PDCD1 transcript levels from fresh tumor biopsies at baseline and cycle 2 or 3 of pembrolizumab treatment. Peripheral blood CD8 (left) and CD4 (right) T cell PD-1 occupancy at baseline and weeks three and six post-treatment with pembrolizumab (A). PDCD1 (PD1) transcript abundance in tumor is unchanged after six weeks (two cycles; left) and nine weeks (three cycles; right) of pembrolizumab treatment (B). Paired tumor biopsies are connected by line. (JPG 867 kb) [file 40425_2019_541_MOESM6_ESM.jpg]

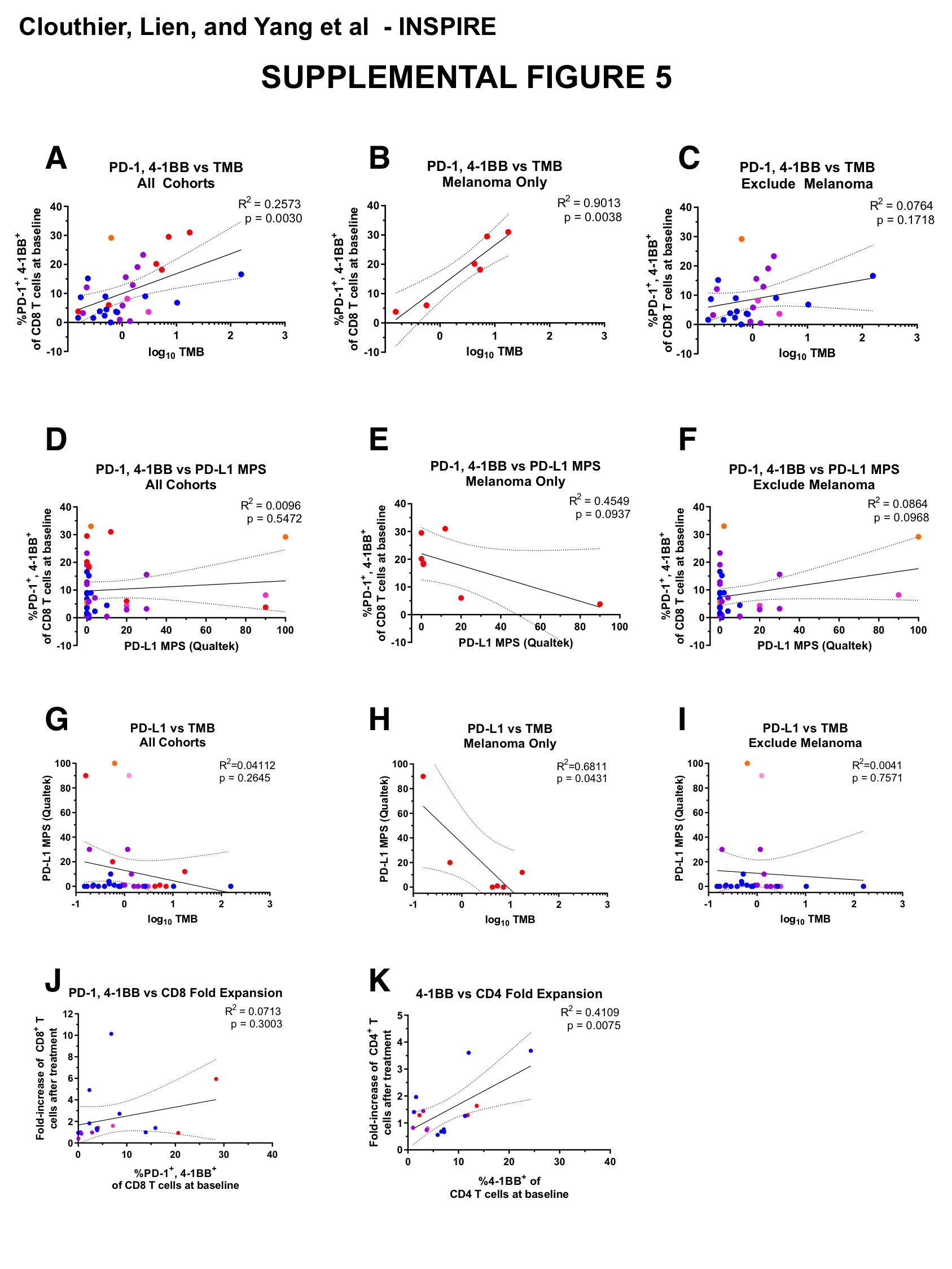

Supplement: Supplementary file 7 — Figure. S5 Pearson correlations between biologically relevant candidate biomarkers. Correlation of TMB and tumor PD-1+ 4-1BB+ CD8 T cells at baseline (A) in all patients, (B) in MM patients, (C) in all patients except MM; PD-L1 MPS and tumor PD-1+ 4-1BB+ CD8 T cells at baseline (D) in all patients, (E) in MM patients, (F) in all patients except MM; TMB and PD-L1 MPS at baseline (G) in all patients, (H) in MM patients, (I) in all patients except MM; and tumor PD-1+ 4-1BB+ CD8 T cells at baseline with fold-expansion of (J) tumor CD8 T cells and (K) tumor CD4 T cells. Orange, SCCHN; Pink, TNBC; Purple, HGSC; Red, MM; Blue, MST. (JPG 446 kb) [file 40425_2019_541_MOESM7_ESM.jpg]

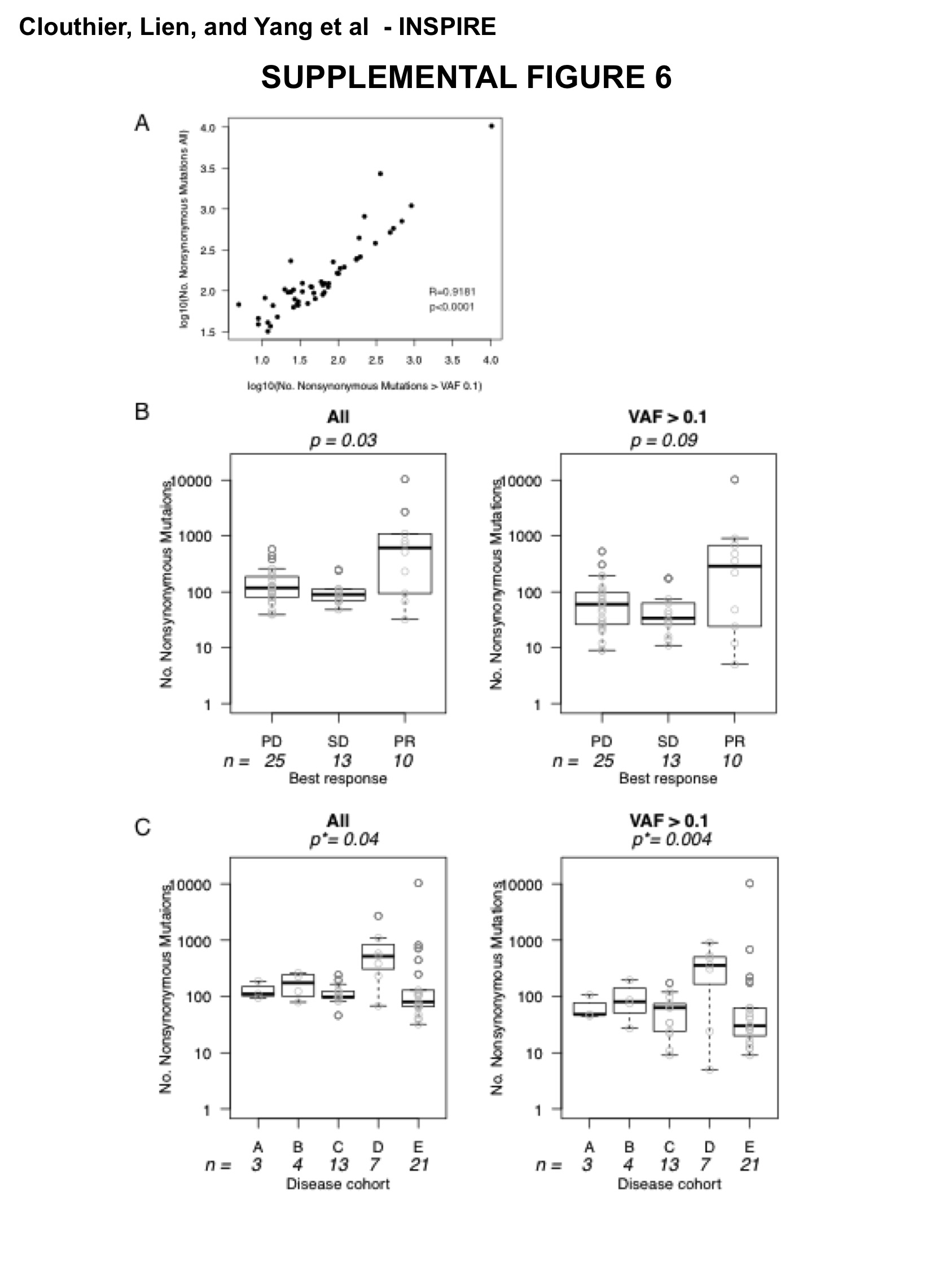

Supplement: Supplementary file 8 — Figure S6. Effect of filtering and tumour type on assessing TMB by exome sequencing. (A) Comparison of total number of non-synonymous mutations detected in each pre-treatment tumour sample (N = 50) with or without minimum variant allele fraction (VAF) threshold applied (VAF > 10%). Pearson correlation and raw p-value for correlation test are show. (B) Boxplot comparison of pre-treatment TMB (total number of non-synonymous mutations) between clinical response groups by RECIST1.1 with or without VAF filter. Statistical significance evaluated using one-way ANOVA. (C) Boxplot comparison of pre-treatment TMB between disease cohorts. Statistically significant differences in TMB between cohorts A to D was evaluated using one-way ANOVA. Cohort E (mixed solid tumor cohort) was excluded from this comparison Additional file 8: Figure S6. (JPG 238 kb) [file 40425_2019_541_MOESM8_ESM.jpg]
